# Supplementary material for: Origin and evolution of qingke barley in Tibet
Source: Nat Commun. 2018 Dec 21;9:5433. doi: 10.1038/s41467-018-07920-5 (PMC6303313; doi:10.1038/s41467-018-07920-5)
Supplement: Supplementary file 1 — Supplementary Information [file 41467_2018_7920_MOESM1_ESM.docx]

**Origin and evolution of qingke barley in Tibet**

Zeng *et al.*


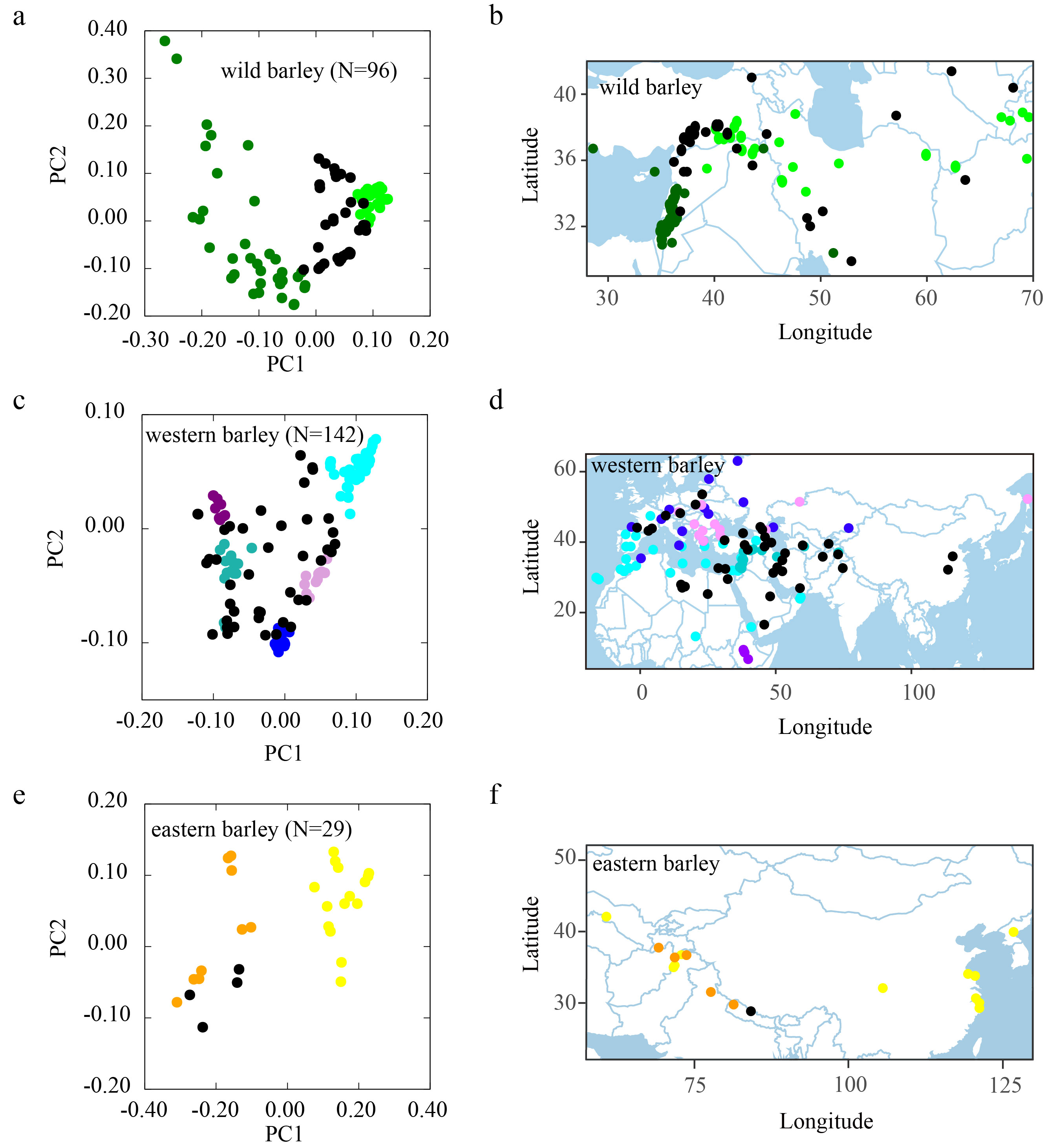


**Supplementary Figure 1. Relationship between population genetic structures and geography.** (a, c, e) Principal components analyses evidenced the subpopulations of wild barley, western barley and eastern barley revealed by sNMF of *K*=9. (b, d, f) The geographical position of each barley accessions. Only wild and landrace accessions which had clear geographical information (longitude and latitude) were plotted. Dots of different color represented the different subpopulations revealed by sNMF of *K*=9. The black dots indicated the admixed accessions. (b, d, f) were generated in R (V3.4.3) using packages maps, maptools, plyr, ggrepel and ggplot2. Geographic information data were obtained from Global Administrative Areas database (GADM V2.8, November 2015). Source data are provided as a Source Data file.


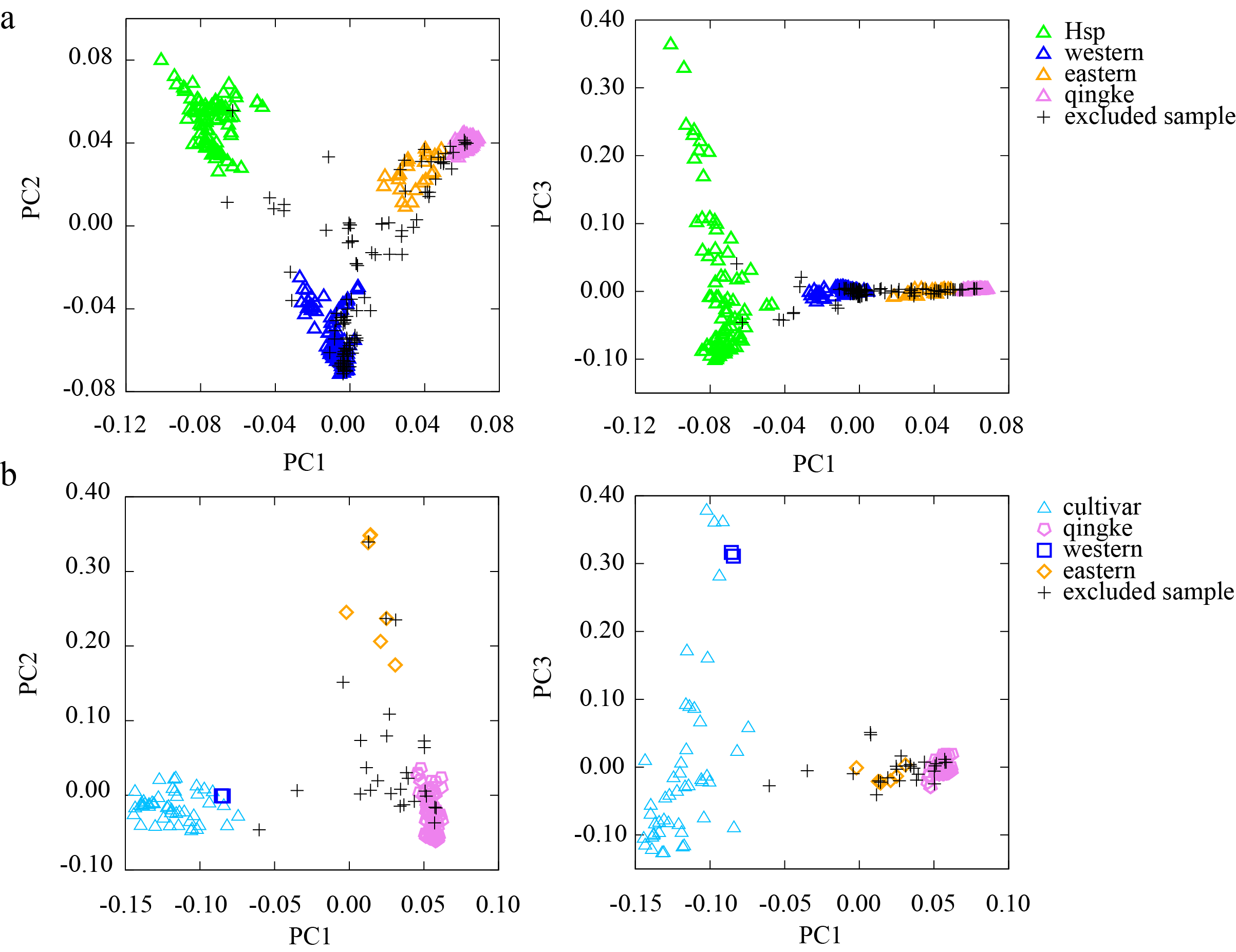


**Supplementary Figure 2. Barley groups based on principal components analyses.** (a) Principal components analyses based on the overlapped SNPs data. The samples marked with excluded sample included (i) the *H. agriocrithon*, cultivars, Tibetan weedy barleys and (ii) samples admixed with other groups. The remainder samples were clustered wild (Hsp: *H.spontaneum*) group and three landrace background groups: western group, eastern group and qingke group. (b) Principal components analyses based on WGS SNPs data. The samples marked excluded sample included (i) the Tibetan weedy barleys (ii) samples admixed with other groups. The remainder samples were clustered three groups: western cultivar groups, eastern Asian groups and qingke groups. The two landraces (Hiproly) collected from Ethiopia marked with western were clustered to the western cultivar groups. Source data are provided as a Source Data file.


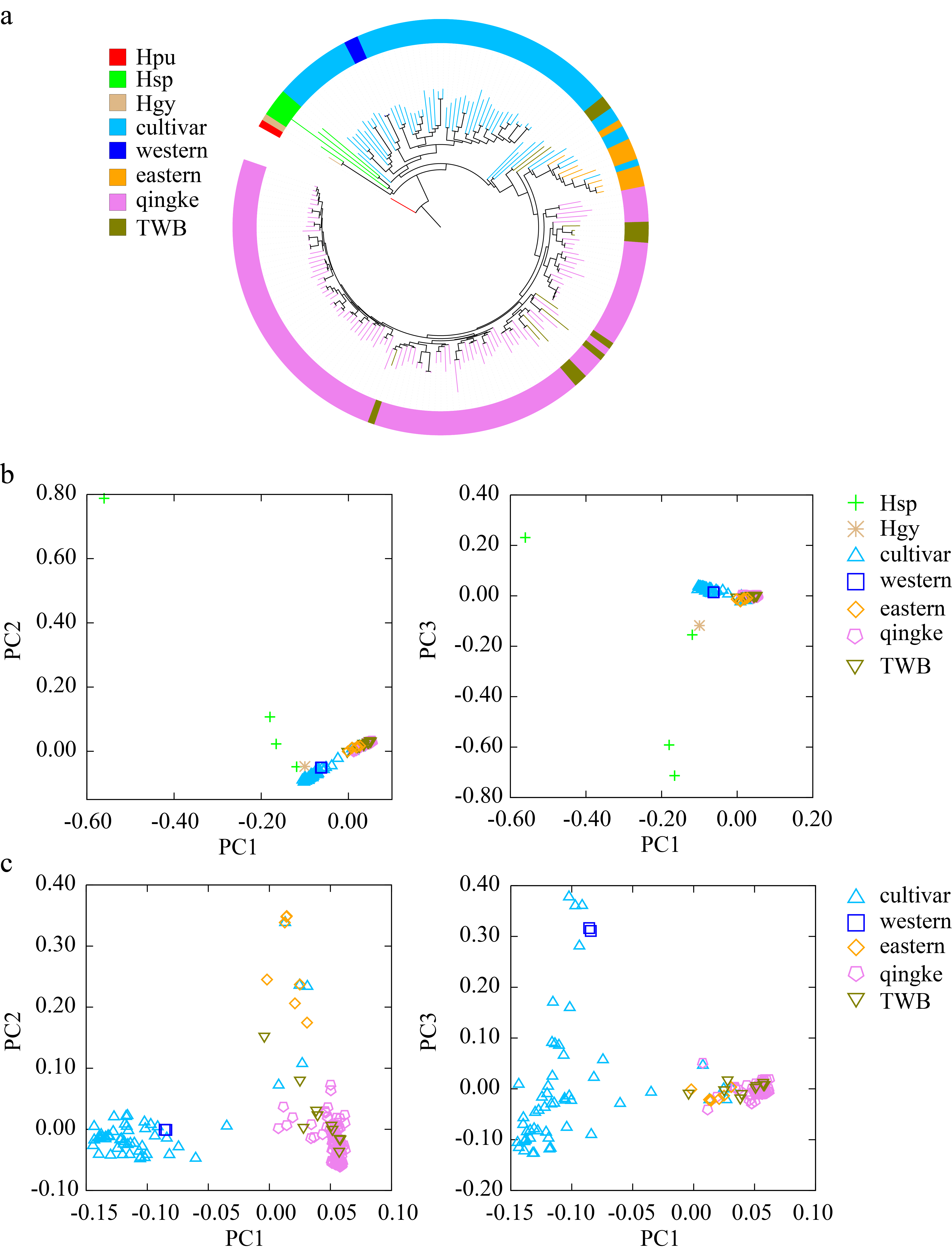


**Supplementary Figure 3. Population structuring based on WGS SNPs data.** (a) Neighbor-joining clustering based on genetic distance. (b) Principal components analyses. (c) Principal components analyses of WGS barley samples without Hsp and Hgy. Removing the 5 wild barley accessions (including semi-wild accessions Hgy) was to improve the PCA resolution for the domesticated barley. Hpu, *H.pubiﬂorum*; Hsp, *H.spontaneum*; Hgy, *H. var. gymnospermum*; cultivar, barley cultivars produced by artificial breeding and selection; western, the two barley landraces (Hiproly) collected from Ethiopia; eastern, the 7 barley landraces collected from Central and eastern China; TWB, Tibetan weedy barley. Source data are provided as a Source Data file.


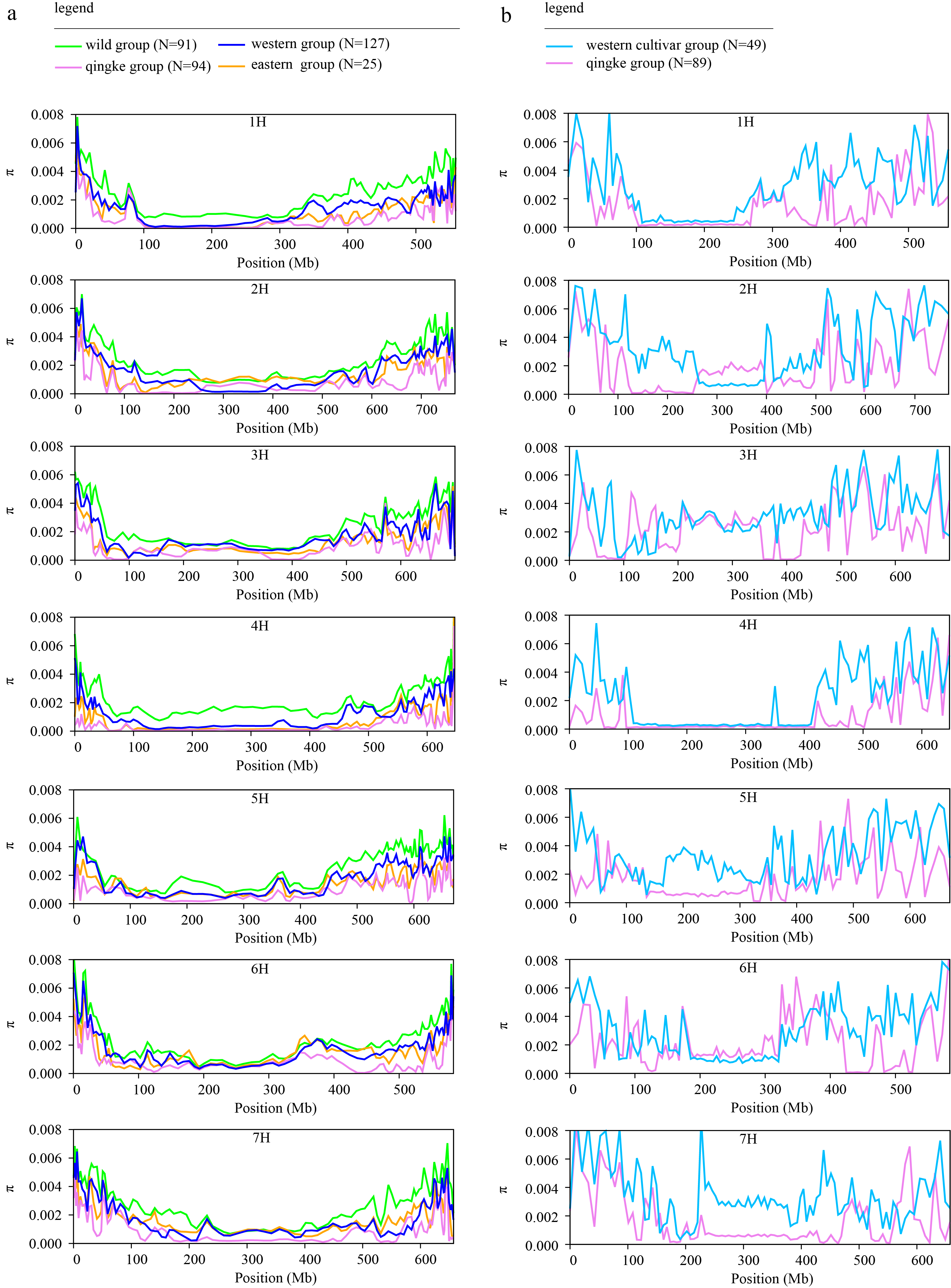


**Supplementary Figure 4. Distribution of nucleotide diversity (*π*) in barley groups.** The unbiased nucleotide diversity (y axis) was calculated for 10 kb window with a step size of 2 kb and plotted with “smooth bezier” treatment of Gnuplot (<http://www.gnuplot.info/>). For improved visualization, all chromosomes have been normalized to a standard length. (a) Distribution of nucleotide diversity on seven chromosomes of four barley groups based on the overlapped SNPs data. (b) Distribution of nucleotide diversity on seven chromosomes of two barley groups based on the WGS SNPs data. Qingke showed lowest genome-wide nucleotide diversity than other barley groups. Source data are provided as a Source Data file.


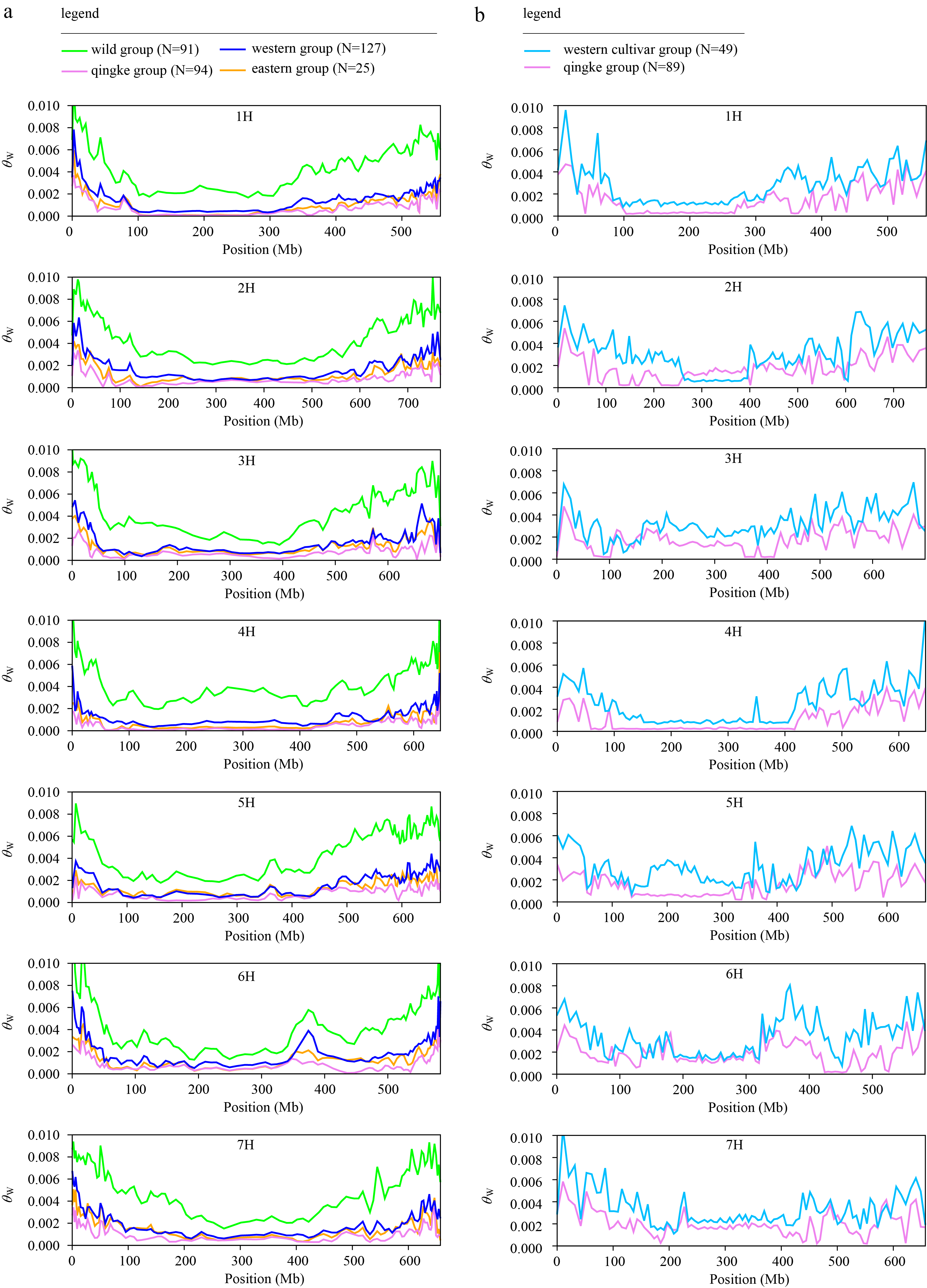


**Supplementary Figure 5. Distribution of Watterson’s estimator (*θ*_W_) in barley groups.** The unbiased *θ*_W_ (y axis) was calculated for 10 kb window with a step size of 2 kb and plotted with “smooth bezier” treatment of Gnuplot (<http://www.gnuplot.info/>). For improved visualization, all chromosomes have been normalized to a standard length. (a) Distribution of *θ*_W_ on seven chromosomes of four barley groups based on the overlapped SNPs data. (b) Distribution of *θ*_W_ on seven chromosomes of two barley groups based on the WGS SNPs data. Profiles reveal an excess counts of polymorphic SNPs sites in wild barley compared to domesticated barley. Qingke showed the lowest level of counts of polymorphic SNPs sites than other barleys. Source data are provided as a Source Data file.


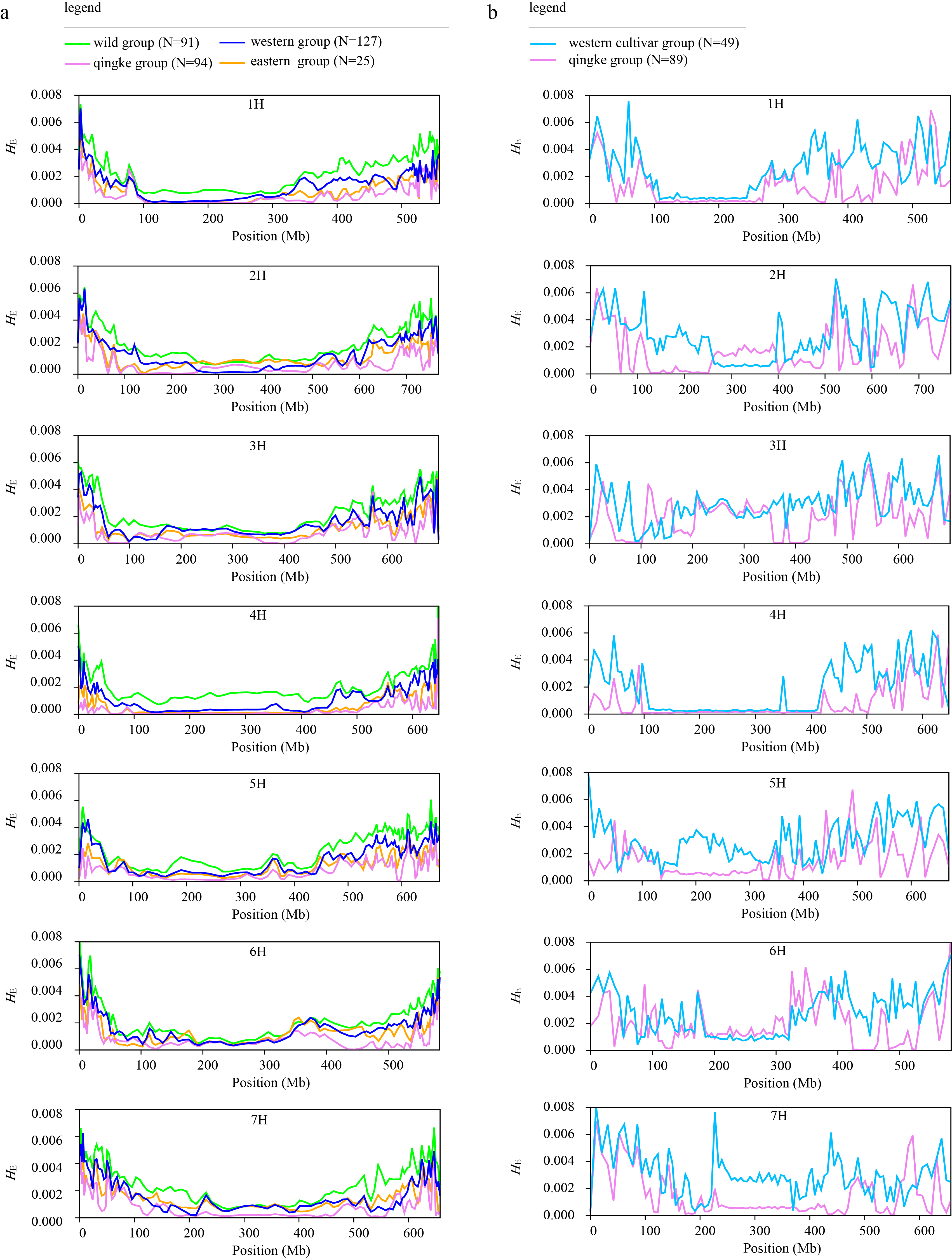


**Supplementary Figure 6. Distribution of Gene diversity/heterozygosity (*H*_E_) in barley groups.** The unbiased *H*_E_ (y axis) was calculated for 10 kb window with a step size of 2 kb and plotted with “smooth bezier” treatment of Gnuplot (<http://www.gnuplot.info/>). For improved visualization, all chromosomes have been normalized to a standard length. (a) Distribution of *H*_E_ on seven chromosomes of four barley groups based on the overlapped SNPs data. (b) Distribution of *H*_E_ on seven chromosomes of two barley groups based on the WGS SNPs data. Profiles reveal an excess of diversity in wild barley compared to domesticated barley. Qingke showed the lowest level than other barleys. Source data are provided as a Source Data file.


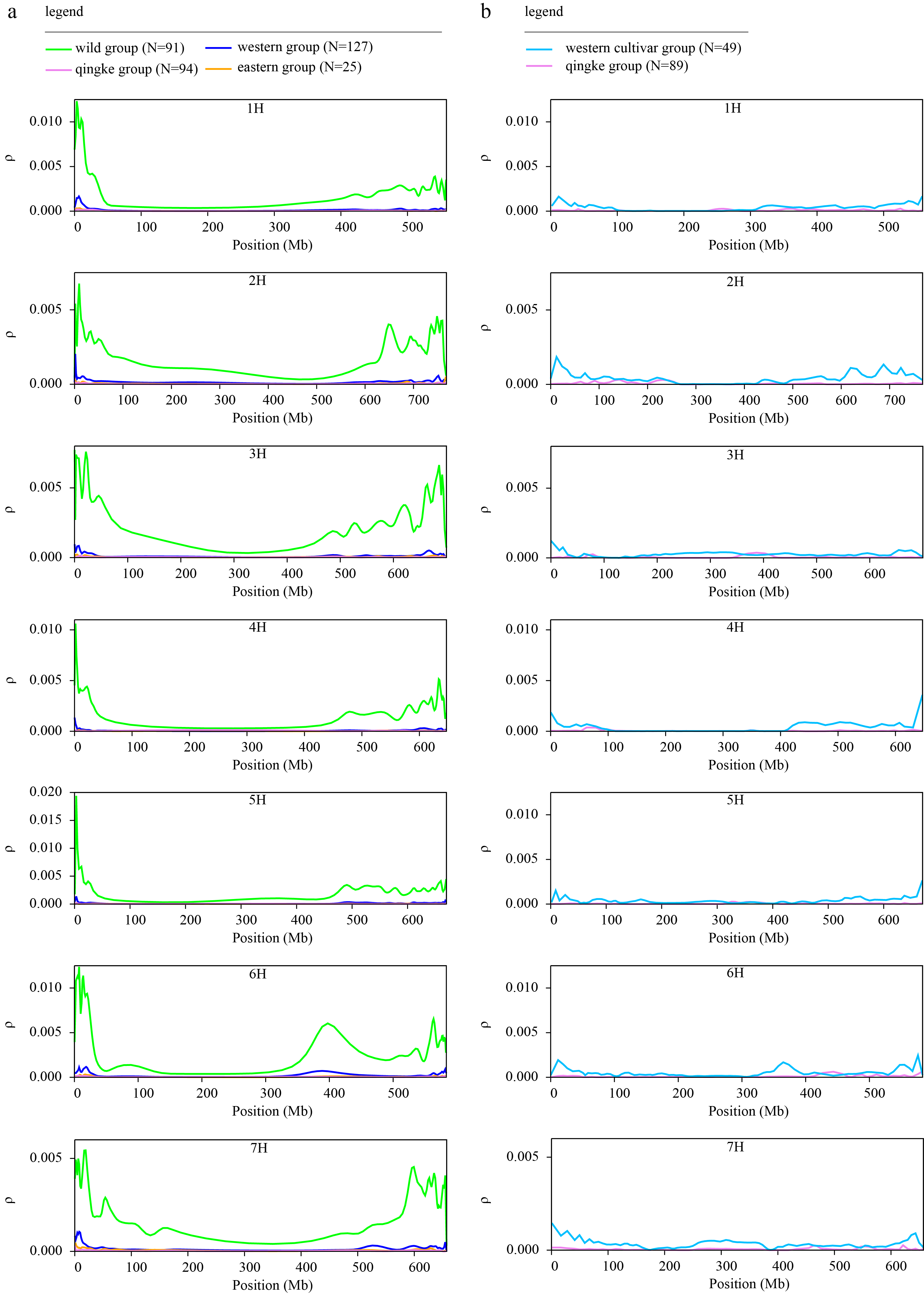


**Supplementary Figure 7. Distribution of recombination rate (*ρ*=4*N_e_r*) in barley groups.** Value was determined with all of the SNPs on contigs and plotted with “smooth bezier” treatment of Gnuplot (<http://www.gnuplot.info/>). For improved visualization, all chromosomes have been normalized to a standard length. (a) Distribution of *ρ* on seven chromosomes of four barley groups based on the overlapped SNPs data. (b) Distribution of *ρ* on seven chromosomes of two barley groups based on the WGS SNPs data. Profiles reveal an excess of recombination rate in wild barley compared to domesticated barley. Qingke showed the lowest level of recombination rate. Source data are provided as a Source Data file.


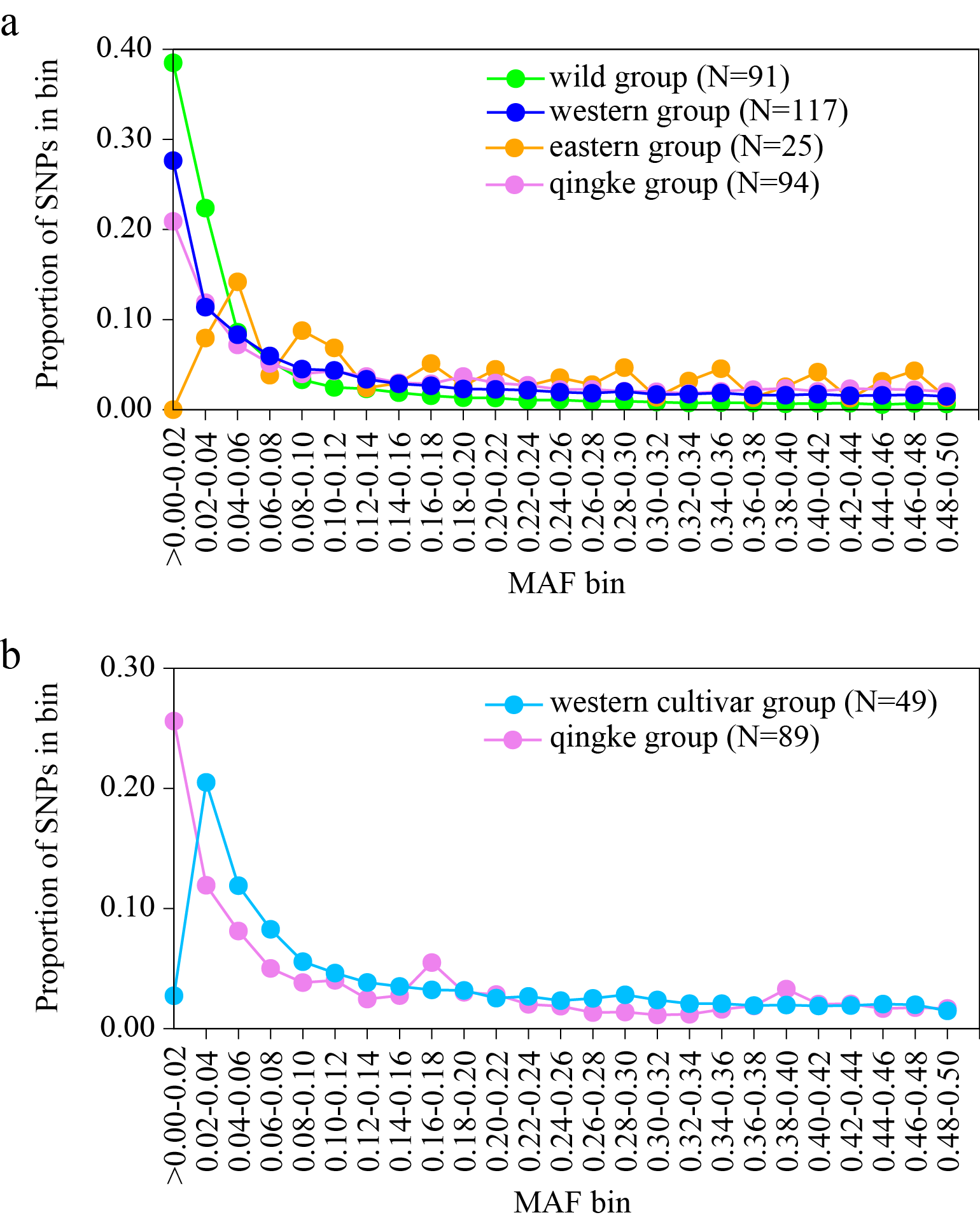


**Supplementary Figure 8. Minor allele frequency (MAF) distributions of SNPs in barley groups.** The non-polymorphic SNPs site (MAF=0) in each barley group were not used. (a) Distributions in four barley groups based on overlapped SNPs data. Profiles reveal an excess of low-frequency SNPs in wild barley compared to domesticated barley. Qingke showed the lowest level of low-frequency SNPs. (b) Distributions in two barley groups based on the WGS SNPs data. Qingke showed the lower level of low-frequency SNPs on the general trend than barley cultivars. Source data are provided as a Source Data file.


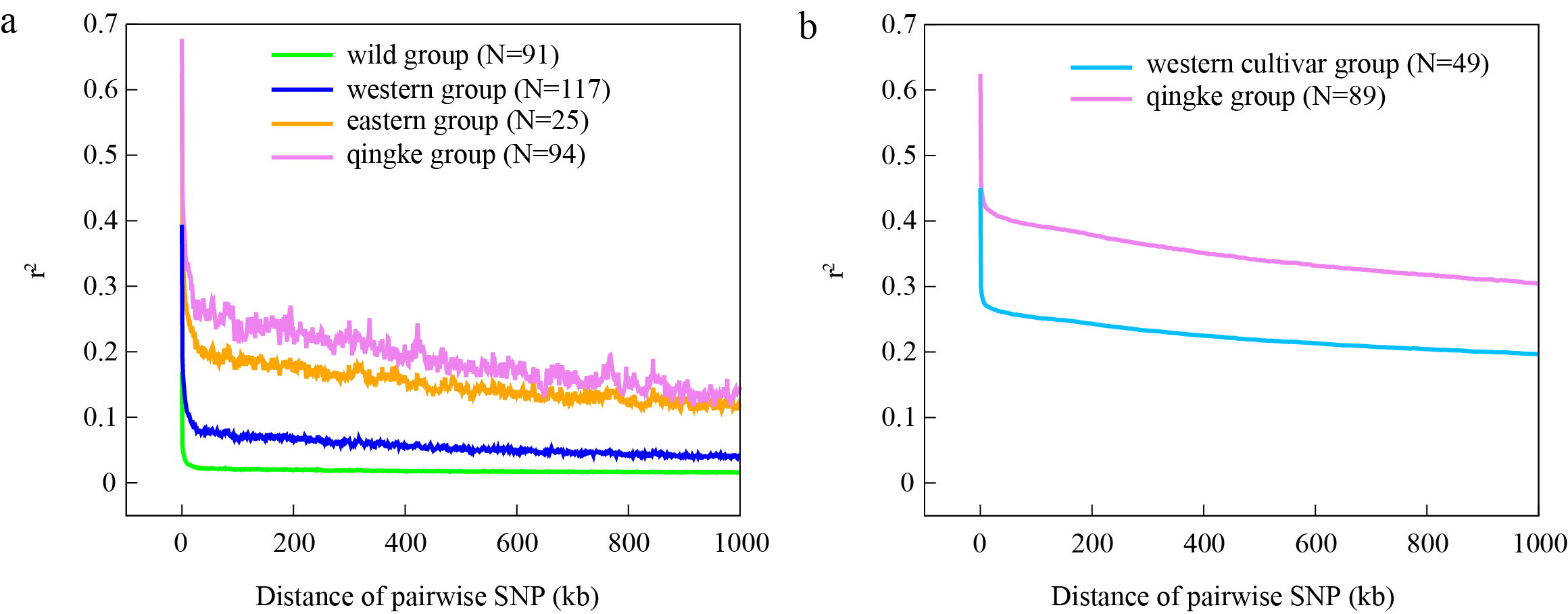


**Supplementary Figure 9. Linkage disequilibrium (*r^2^*) differences among barley groups.** Values were determined for all pairs of SNPs within 1Mb and plotted as a function of distance between SNP pairs. (a) Linkage disequilibrium in four barley groups based on the overlapped SNPs data. Estimates decay more rapidly and reach a lower basal level for wild barley. Qingke showed the highest linkage disequilibrium level. (b) Linkage disequilibrium in two barley groups based on the WGS SNPs data. Qingke showed the higher linkage disequilibrium level than barley cultivars. Source data are provided as a Source Data file.

**
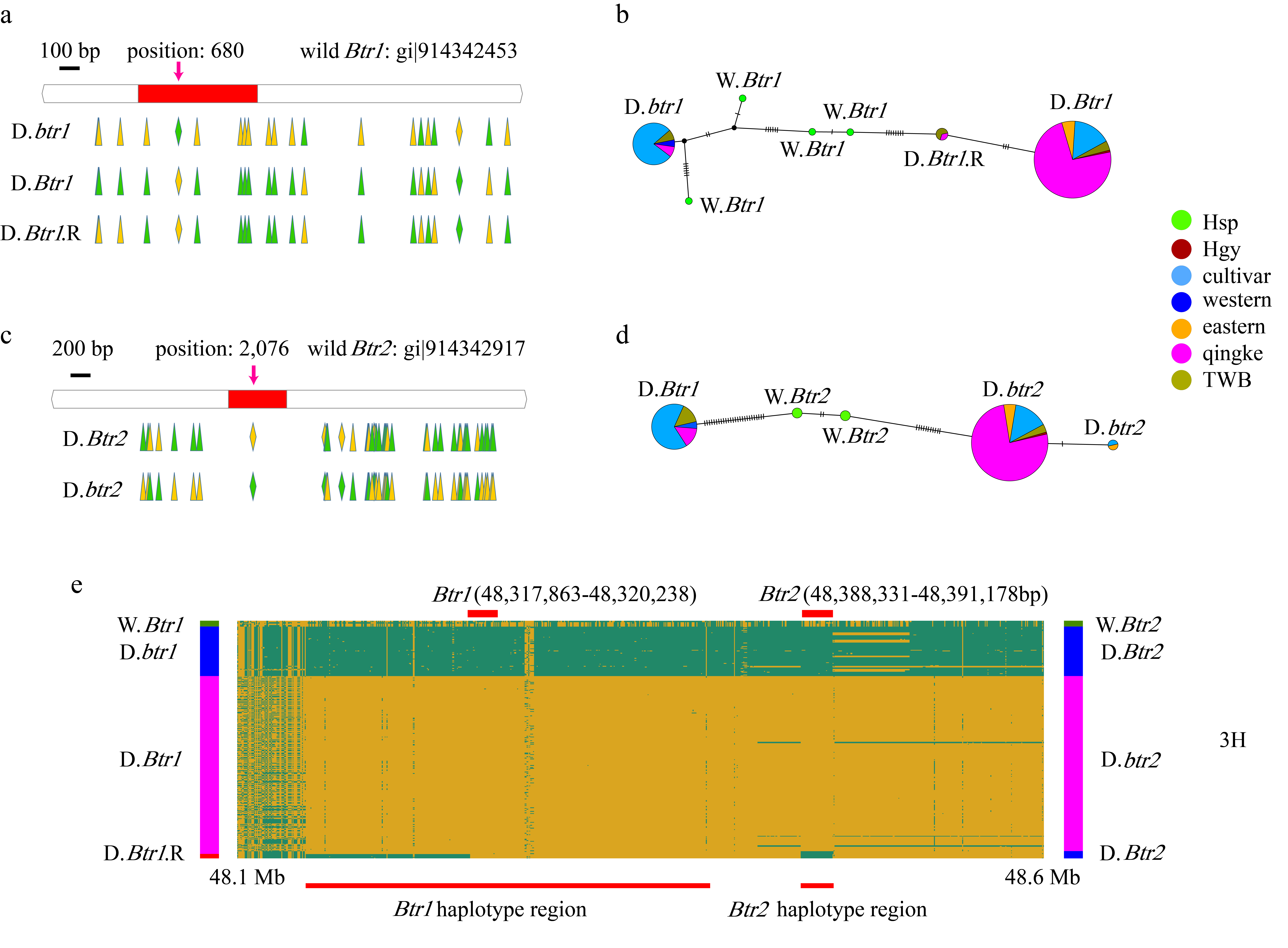
**

**Supplementary Figure 10. Molecular variation in *Btr1* and *Btr2* of 177 WGS barley samples.** W.*Btr1*: wild-type alleles *Btr1*; W.*Btr2*: wild-type alleles *Btr2*; D.*Btr1*: domesticated-type alleles *Btr1*; D.*btr1*: domesticated-type alleles *btr1*; D.*Btr2*: domesticated-type alleles *Btr2*; D.*btr2*: domesticated-type alleles *btr2*; D.*Btr1*.R: the recombined-type alleles with D.*Btr1* and D.*Btr2*. (a, c) Gene structures (exon: red bar) of *Btr1* (a) and *Btr2* (c) with the relative positions of the SNPs (triangle) and INDELs (rhombus) indicated, respectively. The golden triangle or rhombus indicated the same genotype compared with the reference gene sequences (wild-type *Btr1* and *Btr2* sequences); and the lime green indicated different. The *btr1* and *btr2* alleles differ from their wild-type alleles *Btr1* and *Btr2* by a 1-bp (*GC*/*G-*) and an 11-bp deletion (*GGCAACGTCTTC*/*G-----------*) respectively located in the coding sequences was showed in the position 680 for *Btr1* (a) and in the position 2,076 for *Btr2* (c). (b, d) Median-joining networks for *Btr1* (b) and *Btr2* (d) haplotypes of 177 WGS barley samples. Hsp: *H. spontaneum*; Hgy: *H.* var*. gymnospermum Korn*; cultivar: barley cultivars; western, western barley landraces; eastern, eastern barley landraces; qingke: qingke barley; TWB: Tibetan weedy barley. (e) The degree of haplotype of 177 WGS samples around the *Btr1* and *Btr2* locations in barley genome. The golden region of corresponding accessions indicated the same genotype compared with the barley genome (genotype of Morex); and the green region indicated different. Source data are provided as a Source Data file.


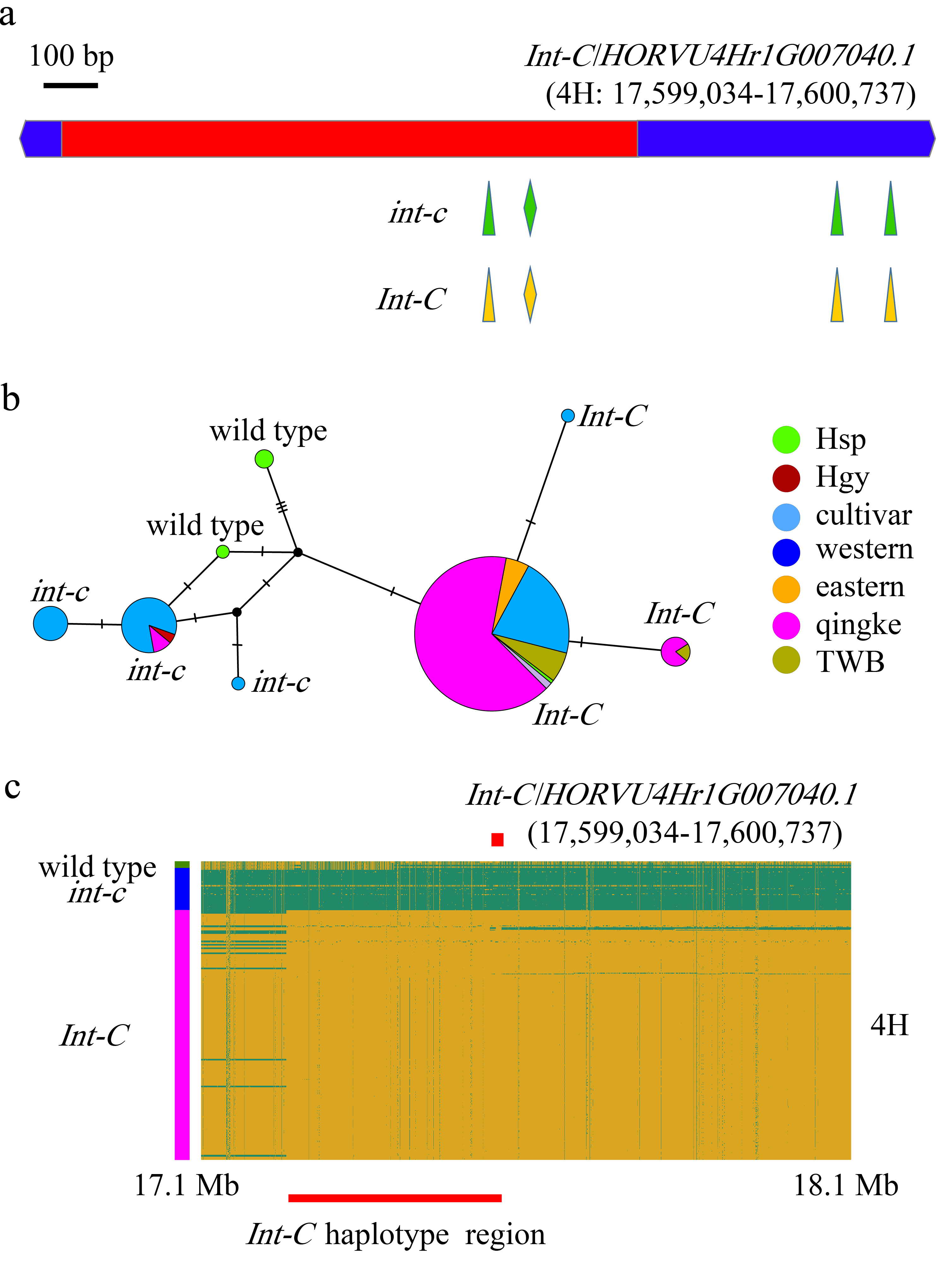


**Supplementary Figure 11. Molecular variation in *Int-C/HORVU4Hr1G007040.1* of 177 WGS barley samples.** (a) Genomics structures (exon: red bar; UTR: blue bar) of *Int-C* with the relative positions of the SNPs (triangle) and INDELs (rhombus) indicated, respectively. The golden triangle or rhombus indicated the same genotype compared with the reference gene sequences (Morex); and the lime green indicated different. (b) Median-joining networks for *Int-C* haplotypes of 177 WGS barley samples. Hsp: *H. spontaneum*; Hgy: *H.* var*. gymnospermum Korn*; cultivar: barley cultivars; western, western barley landraces; eastern, eastern barley landraces; qingke: qingke barley; TWB: Tibetan weedy barley. (c) The degree of haplotype of 177 WGS samples around the *Int-C* locations in barley genome. The golden region of corresponding accessions indicated the same genotype compared with the barley genome (genotype of Morex); and the green region indicated different. Source data are provided as a Source Data file.


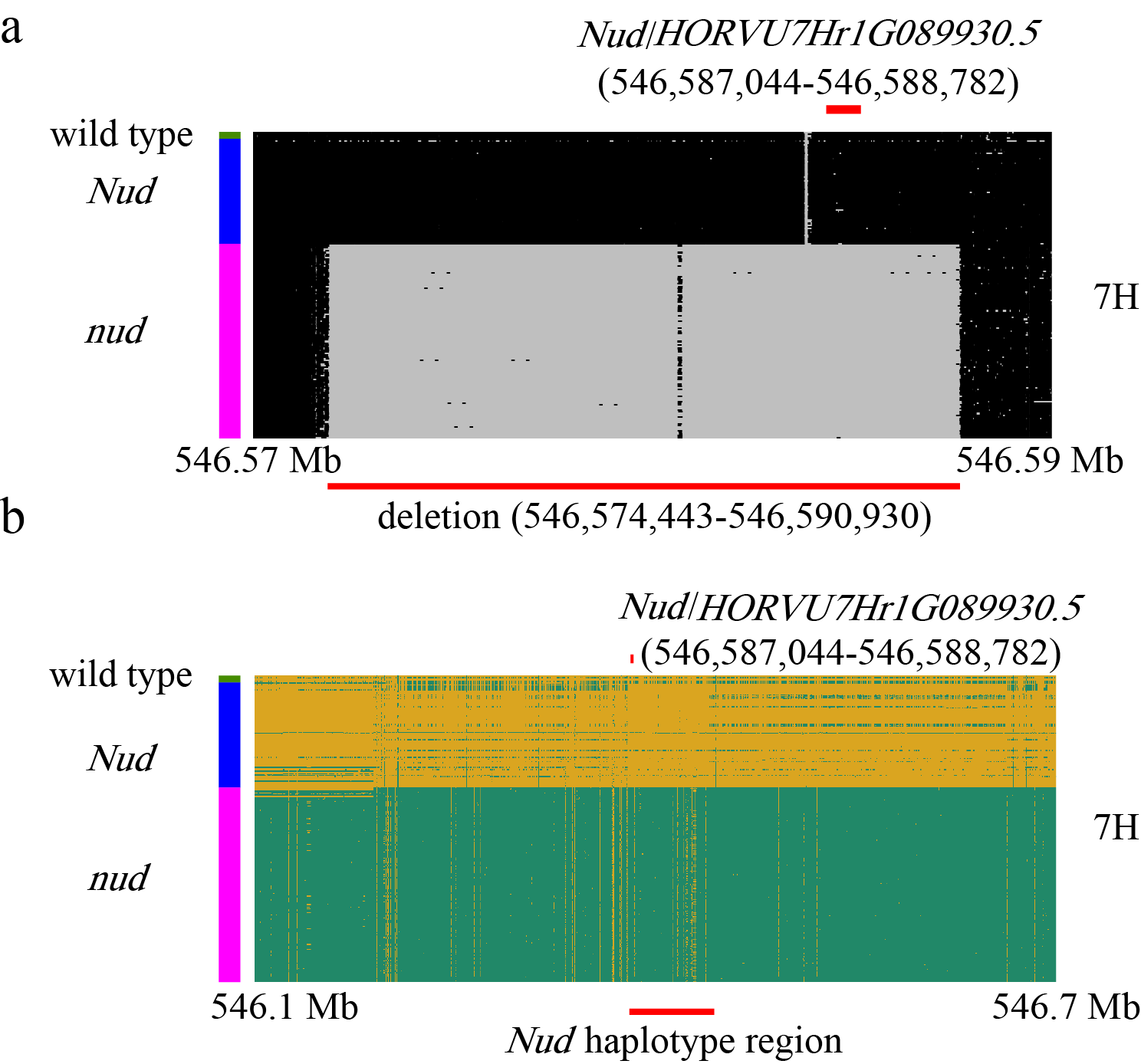


**Supplementary Figure 12. Molecular variation in *Nud/HORVU7Hr1G089930.5* of 177 WGS barley samples.** (a) The reads depth of 177 WGS barley samples around the *Nud* location in barley genome. The black region indicated the reads depth were ≥1× in the region of corresponding accessions. The gray region indicated no reads covered, deletions regions compared to barley genome. (b) The degree of haplotype of 177 WGS samples around the *Nud* locations in barley genome. The golden region of corresponding accessions indicated the same genotype compared with the barley genome (genotype of Morex); and the green region indicated different. Source data are provided as a Source Data file.


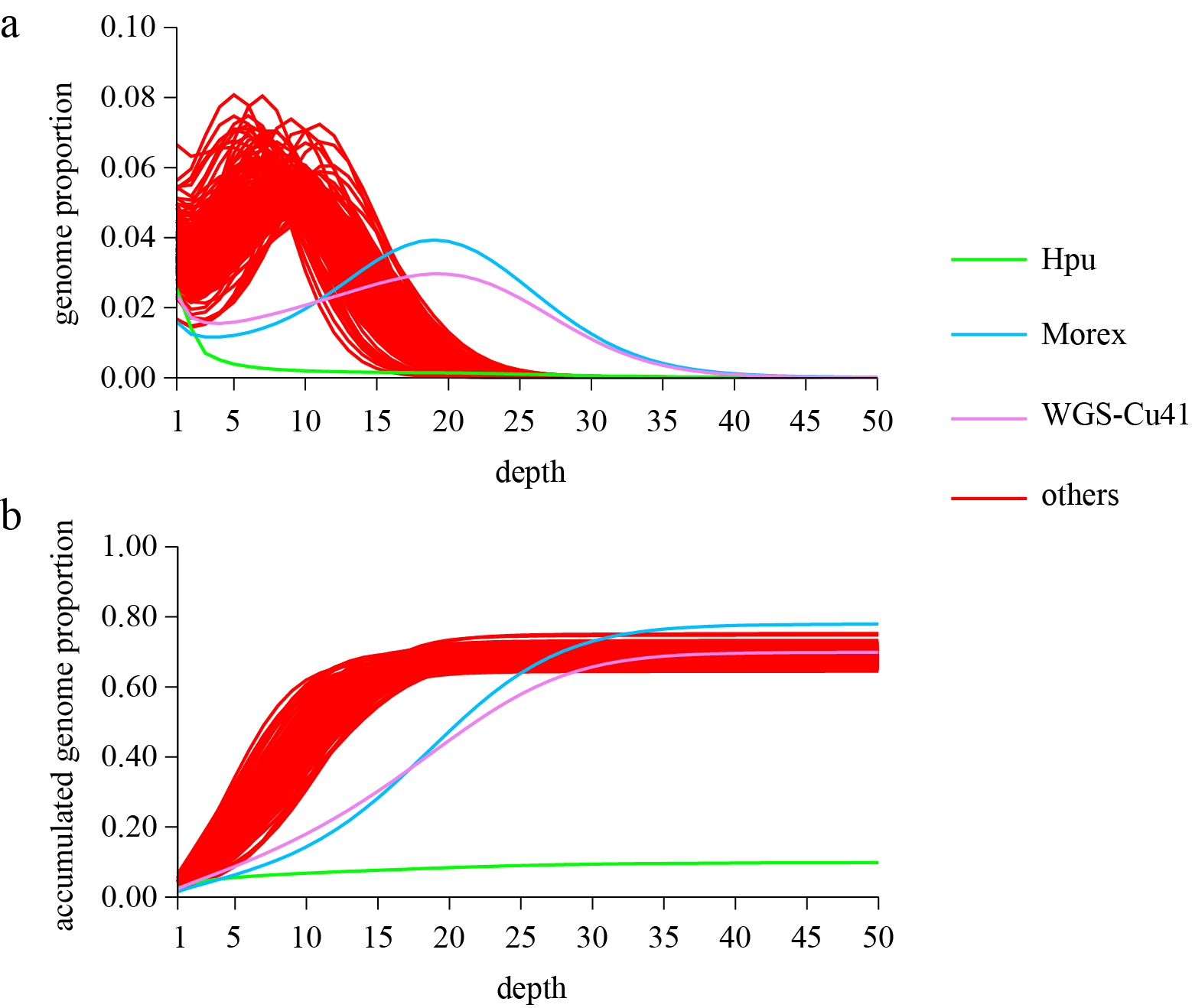


**Supplementary Figure 13. Proportion of barley genome (a) and accumulated proportion of barley genome (b) covered by different read depths of each accession.** Only high quality mapped reads (mapped, non-duplicated reads with mapping quality ≥20) were used for the statistics. Hpu: *H. pubiﬂorum*; Morex (WGS-Cu4) and WGS-Cu41 were the deeply sequenced accessions (≥20×); others meant the other barley accessions with ~9.6× coverage fold. Source data are provided as a Source Data file.


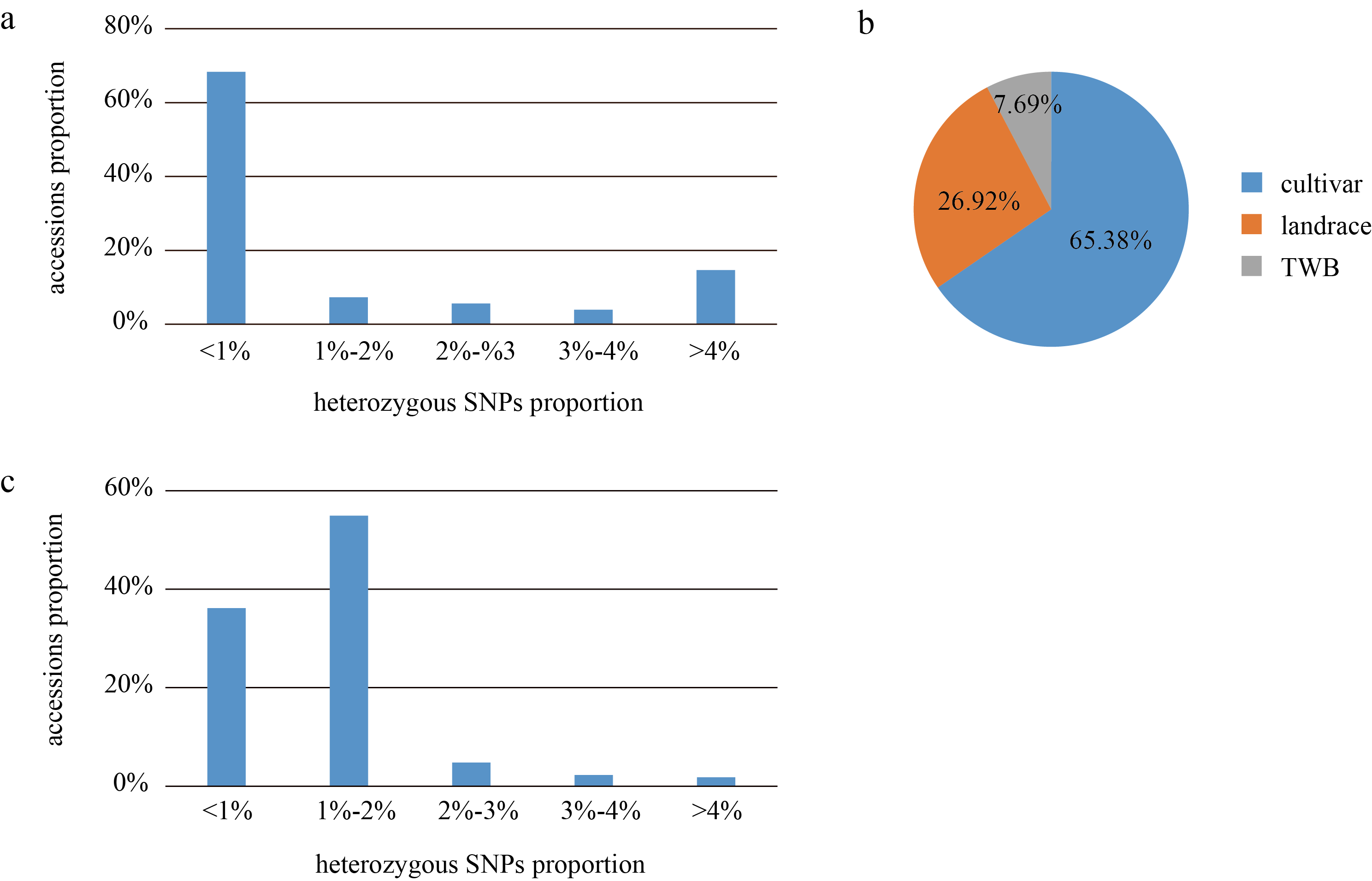


**Supplementary Figure 14. Proportion of accessions with different heterozygous SNPs proportions in the WGS SNPs data (a) and overlapped SNPs data (c).** The heterozygous SNPs proportions of each accessions were equals to the counts of heterozygous SNPs divided by the total counts of SNPs site. (b) The proportion of cultivars, landraces and TWB (Tibetan weedy barley) in the 26 accessions which had >4% heterozygous SNPs proportion. Source data are provided as a Source Data file.


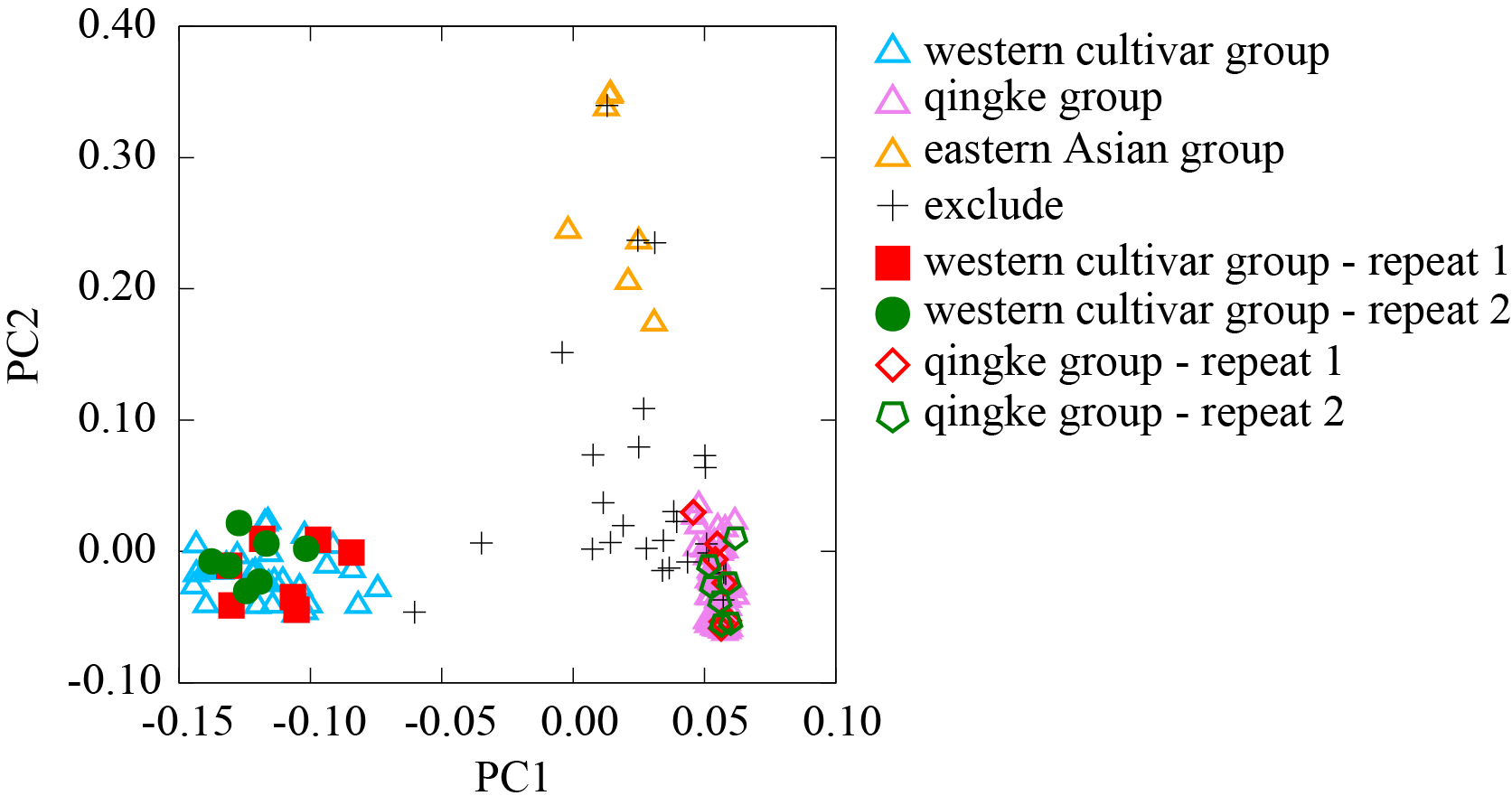


**Supplementary Figure 15. PCA state of the WGS accessions chosen for SMC++ running.** The PCA profile was the same showed in Supplementary Figure 2b. We randomly selected 7 even-distributed accessions in PCA profile form western cultivar group and qingke group, respectively, keeping the same sample size with eastern Asia group. The random selection was repeated for 2 times for western cultivar group and qingke group. The exclude indicated the accessions were removed from WGS barley groups. Source data are provided as a Source Data file.

**Supplementary Table 1. Distribution of Tibetan population (Zang people) in Tibet and Tibetan barley (qingke and Tibetan weedy barley) used in this study.**

| region | counts of Tibetans  (Zang people) | counts of qingke landrace | counts of Tibetan weedy barley |
| --- | --- | --- | --- |
| Ngari | 95,465 | 3 | 0 |
| Rikaze | 703,292 | 14 | 2 |
| Naqu | 462,382 | 9 | 2 |
| Lasa | 559,423 | 5 | 0 |
| Shannan | 328,990 | 10 | 2 |
| Linzhi | 195,109 | 9 | 2 |
| Changdu | 657,505 | 10 | 2 |

The distribution of Tibetan population was based on the official census in 2010 (http://www.stats.gov.cn/tjsj/tjgb/rkpcgb/dfrkpcgb/201202/t20120228_30406.html).

**Supplementary Table 2. Statistics of high confidence SNPs and INDELs data in barley chromosomes.**

| chromosome | WGS SNPs data | WGS INDELs data | overlapped SNPs data |
| --- | --- | --- | --- |
| 1H | 6,382,697 | 455,793 | 189,245 |
| 2H | 9,317,795 | 648,479 | 242,166 |
| 3H | 9,032,898 | 630,621 | 235,374 |
| 4H | 7,580,897 | 497,354 | 195,255 |
| 5H | 7,377,821 | 537,555 | 235,129 |
| 6H | 7,802,621 | 506,224 | 183,839 |
| 7H | 8,397,446 | 595,147 | 238,875 |
| chrUn | 457,184 | 42,283 | 26,070 |
| total | 56,349,359 | 3,913,456 | 1,545,953 |

**Supplementary Table 3. Annotation of the high confidence SNPs and INDELs data by the gene sets of barley genome.**

|  | WGS SNPs data | |  | WGS INDELs data | |
| --- | --- | --- | --- | --- | --- |
|  | HC* | LC* |  | HC* | LC* |
| cds | 0.54% | 0.35% |  | 0.34% | 0.43% |
| 5'utr | 0.15% | 0.31% |  | 0.36% | 0.50% |
| 3'utr | 0.34% | 0.39% |  | 0.75% | 0.66% |
| intron | 1.66% | 0.48% |  | 3.12% | 0.69% |
| stream* | 2.32% | 2.24% |  | 4.78% | 3.59% |
| intergenic | 91.21% | |  | 84.78% | |
| nonsy/sy* | 1.11 | 2.53 |  |  |  |
| Frameshift |  |  |  | 0.23% | 0.35% |

*HC: high confidence genes; LC: low confidence genes

*stream: upstream and downstream ±1kb of exons

*nonsy/sy: counts of nonsynonymous SNPs divided by counts of synonymous SNPs

**Supplementary Table 4. Nucleotide diversity (*π*, 1×10^-3^) in barley groups. Values of *π* are reported per bp.**

|  | overlapped SNPs data | | | |  | WGS SNPs data | |
| --- | --- | --- | --- | --- | --- | --- | --- |
|  | wild | western | eastern | qingke |  | western cultivars | qingke |
| 1H | 3.07 | 1.88 | 1.52 | 1.11 |  | 2.49 | 1.21 |
| 2H | 3.06 | 2.18 | 1.92 | 1.15 |  | 2.87 | 1.54 |
| 3H | 3.08 | 2.18 | 1.81 | 1.26 |  | 3.22 | 2.15 |
| 4H | 2.70 | 1.56 | 1.03 | 0.53 |  | 1.81 | 0.65 |
| 5H | 3.09 | 2.19 | 1.81 | 1.18 |  | 3.11 | 1.58 |
| 6H | 2.84 | 2.13 | 1.91 | 1.18 |  | 2.76 | 1.94 |
| 7H | 3.35 | 2.22 | 2.16 | 1.25 |  | 3.24 | 1.42 |
| chrUn | 2.73 | 1.81 | 1.61 | 1.11 |  | 1.98 | 1.35 |
| all* | 3.03 | 2.06 | 1.75 | 1.10 |  | 2.77 | 1.49 |

*all: all of the chromosomes

Source data are provided as a Source Data file.

**Supplementary Table 5. Watterson’s estimator (*θ*_W_, 1×10^-3^) in barley groups. Values of *θ*_W_ are reported per bp.**

|  | overlapped SNPs data | | | |  | WGS SNPs data | |
| --- | --- | --- | --- | --- | --- | --- | --- |
|  | wild | western | eastern | qingke |  | western cultivars | qingke |
| 1H | 5.42 | 1.92 | 1.36 | 0.98 |  | 2.48 | 1.18 |
| 2H | 5.39 | 2.27 | 1.65 | 1.07 |  | 2.75 | 1.54 |
| 3H | 5.45 | 2.09 | 1.69 | 1.01 |  | 3.07 | 1.62 |
| 4H | 5.09 | 1.54 | 1.02 | 0.64 |  | 2.13 | 0.85 |
| 5H | 5.44 | 2.04 | 1.64 | 1.01 |  | 2.99 | 1.50 |
| 6H | 5.14 | 2.28 | 1.68 | 1.10 |  | 3.09 | 1.79 |
| 7H | 5.79 | 2.32 | 1.90 | 1.20 |  | 3.17 | 1.87 |
| chrUn | 4.91 | 1.91 | 1.45 | 0.94 |  | 2.17 | 1.29 |
| all* | 5.39 | 2.07 | 1.57 | 1.00 |  | 2.79 | 1.47 |

*all: all of the chromosomes

Source data are provided as a Source Data file.

**Supplementary Table 6. Gene diversity/heterozygosity (*H*_E_, 1×10^-3^) in barley groups. Values of *H*_E_ are reported per bp.**

|  | overlapped SNPs data | | | |  | WGS SNPs data | |
| --- | --- | --- | --- | --- | --- | --- | --- |
|  | wild | western | eastern | qingke |  | western cultivars | qingke |
| 1H | 2.88 | 1.76 | 1.30 | 0.98 |  | 2.27 | 1.09 |
| 2H | 2.88 | 2.06 | 1.68 | 1.04 |  | 2.59 | 1.43 |
| 3H | 2.90 | 2.05 | 1.58 | 1.14 |  | 2.94 | 1.98 |
| 4H | 2.55 | 1.48 | 0.88 | 0.47 |  | 1.65 | 0.59 |
| 5H | 2.91 | 2.08 | 1.58 | 1.07 |  | 2.82 | 1.44 |
| 6H | 2.64 | 2.00 | 1.66 | 1.05 |  | 2.51 | 1.79 |
| 7H | 3.14 | 2.09 | 1.89 | 1.12 |  | 2.94 | 1.29 |
| chrUn | 2.60 | 1.73 | 1.44 | 0.99 |  | 1.67 | 1.19 |
| all* | 2.85 | 1.94 | 1.52 | 0.99 |  | 2.51 | 1.36 |

*all: all of the chromosomes

Source data are provided as a Source Data file.

**Supplementary Table 7. Recombination rate (*ρ*=4*N_e_r*, 1×10^-3^) of each barley groups. Values of ρ are reported per bp.**

|  | overlapped SNPs data | | | |  | WGS SNPs data | |
| --- | --- | --- | --- | --- | --- | --- | --- |
|  | wild | western | eastern | qingke |  | western cultivars | qingke |
| 1H | 2.884 | 0.242 | 0.064 | 0.053 |  | 0.320 | 0.074 |
| 2H | 2.551 | 0.235 | 0.069 | 0.041 |  | 0.351 | 0.056 |
| 3H | 3.140 | 0.187 | 0.073 | 0.036 |  | 0.246 | 0.045 |
| 4H | 2.535 | 0.154 | 0.046 | 0.039 |  | 0.226 | 0.046 |
| 5H | 2.720 | 0.206 | 0.057 | 0.038 |  | 0.338 | 0.049 |
| 6H | 3.903 | 0.360 | 0.096 | 0.064 |  | 0.452 | 0.081 |
| 7H | 2.451 | 0.234 | 0.092 | 0.042 |  | 0.315 | 0.053 |
| chrUn | 2.840 | 0.386 | 0.101 | 0.062 |  | 0.459 | 0.073 |
| all* | 2.852 | 0.231 | 0.072 | 0.044 |  | 0.324 | 0.058 |

*all: all of the chromosomes

Source data are provided as a Source Data file.

**Supplementary Table 8. D statistics based on the overlapped SNPs data for different comparisons among wild-WA, wild-CA, western and eastern barley.**

| P_1_ | P_2_ | P_3_ | P_4_ (outgroup) | D | SE | Z |
| --- | --- | --- | --- | --- | --- | --- |
| wild-CA | wild-WA | qingke | *H.pubiflorum* | 0.001861 | 0.000244 | 7.635 |
| wild-CA | western | qingke | *H.pubiflorum* | -0.00697 | 0.000411 | -16.973 |
| wild-CA | eastern | qingke | *H.pubiflorum* | -0.02321 | 0.000438 | -53.046 |
| wild-WA | wild-CA | qingke | *H.pubiflorum* | -0.00186 | 0.000244 | -7.635 |
| wild-WA | western | qingke | *H.pubiflorum* | -0.00883 | 0.000348 | -25.351 |
| wild-WA | eastern | qingke | *H.pubiflorum* | -0.02507 | 0.000428 | -58.61 |
| western | wild-CA | qingke | *H.pubiflorum* | 0.006972 | 0.000411 | 16.973 |
| western | wild-WA | qingke | *H.pubiflorum* | 0.008833 | 0.000348 | 25.351 |
| western | eastern | qingke | *H.pubiflorum* | -0.01624 | 0.000443 | -36.667 |
| eastern | wild-CA | qingke | *H.pubiflorum* | 0.023211 | 0.000438 | 53.046 |
| eastern | wild-WA | qingke | *H.pubiflorum* | 0.025072 | 0.000428 | 58.61 |
| eastern | western | qingke | *H.pubiflorum* | 0.01624 | 0.000443 | 36.667 |

The *P*_3_ was fixed by qingke and *P*_4_ was fixes by *H.pubiflorum*. Standard errors and Z scores (D/SE) are given in columns SE and Z. The wild-CA was the subpopulation of wild barley distributed in central Asia; the wild-WA was the subpopulation of wild barley distributed in western Asia.

**Supplementary Table 9. D statistics based on the overlapped SNPs data for different comparisons among wild barley, western barley, eastern-CA and eastern-EA.**

| P1 | P2 | P3 | P4 (outgroup) | D | SE | Z |
| --- | --- | --- | --- | --- | --- | --- |
| wild | western | qingke | *H.pubiflorum* | -0.00711 | 0.000327 | -21.759 |
| wild | eastern-CA | qingke | *H.pubiflorum* | -0.02662 | 0.000393 | -67.799 |
| wild | eastern-EA | qingke | *H.pubiflorum* | -0.01914 | 0.000421 | -45.448 |
| western | wild | qingke | *H.pubiflorum* | 0.007105 | 0.000327 | 21.759 |
| western | eastern-CA | qingke | *H.pubiflorum* | -0.01951 | 0.000422 | -46.281 |
| western | eastern-EA | qingke | *H.pubiflorum* | -0.01203 | 0.000447 | -26.943 |
| eastern-CA | wild | qingke | *H.pubiflorum* | 0.026616 | 0.000393 | 67.799 |
| eastern-CA | western | qingke | *H.pubiflorum* | 0.019511 | 0.000422 | 46.281 |
| eastern-CA | eastern-EA | qingke | *H.pubiflorum* | 0.007481 | 0.000361 | 20.722 |
| eastern-EA | wild | qingke | *H.pubiflorum* | 0.019136 | 0.000421 | 45.448 |
| eastern-EA | western | qingke | *H.pubiflorum* | 0.012031 | 0.000447 | 26.943 |
| eastern-EA | eastern-CA | qingke | *H.pubiflorum* | -0.00748 | 0.000361 | -20.722 |

The *P*_3_ was fixed by qingke and *P*_4_ was fixes by *H.pubiflorum*. Standard errors and Z scores (D/SE) are given in columns SE and Z. The eastern-CA was the subpopulation of eastern barley distributed in central Asia; the eastern-EA was the subpopulation of eastern barley distributed in eastern Asia.

**Supplementary Table 10. Effective covered region of 177 WGS accessions.**

|  | barley genome | WGS effective covered region | | exome target region | overlapped effective covered region | |
| --- | --- | --- | --- | --- | --- | --- |
|  | size without N (bp) | size (bp) | p1* | size (bp) | size(bp) | p2* |
| 1H | 529,480,960 | 339,026,273 | 64.0% | 6,205,746 | 5,883,725 | 94.8% |
| 2H | 724,295,851 | 418,997,511 | 57.8% | 7,770,275 | 7,358,575 | 94.7% |
| 3H | 675,418,023 | 393,014,835 | 58.2% | 7,662,214 | 7,231,908 | 94.4% |
| 4H | 613,062,221 | 417,465,114 | 68.1% | 6,645,613 | 6,346,193 | 95.5% |
| 5H | 641,949,955 | 345,527,708 | 53.8% | 7,599,205 | 7,197,547 | 94.7% |
| 6H | 547,667,042 | 337,529,550 | 61.6% | 5,999,234 | 5,727,379 | 95.5% |
| 7H | 615,839,859 | 366,134,965 | 59.5% | 7,298,406 | 6,921,109 | 94.8% |
| chrUn | 223,138,410 | 26,870,881 | 12.0% | 989,622 | 879,754 | 88.9% |
| total | 4,570,852,321 | 2,644,566,837 | 57.9% | 50,170,315 | 47,546,190 | 94.8% |

The regions covered by at least two reads in ≥80% of the WGS accessions were defined as the WGS effective covered region of barley genome. The overlapped effective covered region was the overlapped region between the WGS effective covered region and exome target region.

*p1: proportion1- the size of WGS effective covered region divided by the size of corresponding barley genome without N

*p2: proportion2- the size of overlapped effective covered region divided by the size of corresponding exome target region

**Supplementary Table 11. Differentiation (fixation index, *F*_ST_) between pairwise groups based on the overlapped SNPs data.**

|  | wild | western | eastern | qingke |
| --- | --- | --- | --- | --- |
| wild | 0.000 |  |  |  |
| western | 0.202 | 0.000 |  |  |
| eastern | 0.279 | 0.304 | 0.000 |  |
| qingke | 0.398 | 0.400 | 0.204 | 0.000 |

Source data are provided as a Source Data file.
