# Supplementary material for: Origin and evolution of qingke barley in Tibet
Source: Nat Commun. 2018 Dec 21;9:5433. doi: 10.1038/s41467-018-07920-5 (PMC6303313; doi:10.1038/s41467-018-07920-5)
Supplement: Supplementary file 3 — Description of Additional Supplementary Files [file 41467_2018_7920_MOESM3_ESM.pdf]

Description of Additional Supplementary Files

File Name: Supplementary Data 1
Description: The information of the 178 WGS accessions.

File Name: Supplementary Data 2
Description: The information of the 260 ES accessions.

File Name: Supplementary Data 3
Description: The candidate selective region and gene for plateau local adaption of qingke revealed by F_{ST}.

File Name: Supplementary Data 4
Description: The haplotype of 177 WGS accessions in *Btr1* locus.

File Name: Supplementary Data 5
Description: The haplotype of 177 WGS accessions in *Btr2* locus.

File Name: Supplementary Data 6
Description: The haplotype of 177 WGS accessions in *Int-C* locus.

File Name: Supplementary Data 7
Description: The haplotype of 177 WGS accessions in *Nud* locus.

File Name: Supplementary Data 8
Description: The haplotype of 177 WGS and 260 ES accessions in *Vrs1* locus.

File Name: Supplementary Data 9
Description: The sequences of PCR primers for SNPs quality estimation.

File Name: Supplementary Data 10
Description: The genotype comparison between GATK and Sanger sequences.

File Name: Supplementary Data 11
Description: The information of five key domestic genes of barley.
